# Discovery of Mosquito Saliva MicroRNAs during CHIKV Infection

**DOI:** 10.1371/journal.pntd.0003386

**Published:** 2015-01-22

**Authors:** Payal D. Maharaj, Steven G. Widen, Jing Huang, Thomas G. Wood, Saravanan Thangamani

**Affiliations:** 1 Department of Pathology, University of Texas Medical Branch, Galveston, Texas, United States of America; 2 Department of Biochemistry and Molecular Biology, University of Texas Medical Branch, Galveston, Texas, United States of America; 3 Institute for Human Infections and Immunity, University of Texas Medical Branch, Galveston, Texas, United States of America; 4 Center for Tropical Diseases, University of Texas Medical Branch, Galveston, Texas, United States of America; Colorado State University, UNITED STATES

## Abstract

Mosquito borne pathogens are transmitted to humans via saliva during blood feeding. Mosquito saliva is a complex concoction of many secretory factors that modulate the feeding foci to enhance pathogen infection and establishment. Multiple salivary proteins/factors have been identified/characterized that enhance pathogen infection. Here, we describe, for the first time, the identification of exogenous microRNAs from mosquito saliva. MicroRNAs are short, 18–24 nucleotide, non-coding RNAs that regulate gene expression, and are generally intracellular. However, circulating miRNAs have been described from serum and saliva of humans. Exogenous miRNAs have not been reported from hematophagous arthropod saliva. We sought to identify miRNAs in the mosquito saliva and their role in Chikungunya virus (CHIKV) infection. Next generation sequencing was utilized to identify 103 exogenous miRNAs in mosquito saliva of which 31 miRNAs were previously unidentified and were designated novel. Several miRNAs that we have identified are expressed only in the CHIKV infected mosquitoes. Five of the saliva miRNAs were tested for their potential to regulated CHIKV infection, and our results demonstrate their functional role in the transmission and establishment of infection during blood feeding on the host.

## Author Summary

Mosquito saliva contains a complex repertoire of bioactive factors that are secreted into blood feeding site, the skin. Infected mosquitoes transmit pathogens to the host during feeding via saliva. The bioactive factors in mosquito saliva are responsible for modulating host hemostasis, immune defenses and pain/itch responses, and have been implicated to enhance pathogen infection and establishment in the host. In our efforts to identify and characterize salivary immunomodulators that enhance Chikungunya virus (CHIKV) transmission, we have discovered, for the first time, exogenous microRNA in mosquito saliva. MicroRNAs (miRNAs) are short, 18–24 nucleotide, non-coding RNAs that regulate gene expression. Short non-coding RNAs were extracted from the saliva of Chikungunya virus (CHIKV) infected and uninfected *Aedes aegypti* and *Aedes albopictus* saliva, and subjected to Illumina next generation sequencing. Bioinformatic analysis revealed the presence of miRNAs in the mosquito saliva. We have also identified several novel miRNAs that are expressed only during CHIKV infection. Though the functional roles of these miRNAs are yet to be established, our *in-vitro* data from testing 5 miRNAs demonstrate their role in the regulation of CHIKV infection. These miRNAs may play an important role in regulating the establishment of CHIKV infection in the mammalian host during blood feeding.

## Introduction

Mosquitoes are a significant public health concern due to their ability to transmit a variety of emerging and reemerging arboviruses [1,2]. Chikungunya virus (CHIKV) is an excellent example of globalization of a mosquito borne disease, as evident from the CHIKV epidemics in the past seven years [3,4]. Chikungunya virus is an *Alphavirus* belonging to the Togaviridae family and is transmitted predominantly by *Aedes aegypti* and *Aedes albopticus* (www.cdc.gov/ncidod/dvbid/Chikungunya). *Aedes aegypti* and *Ae. albopictus* transmit CHIKV during blood meal acquisition, along with the saliva the mosquitoes inject into the skin. The complex repertoire of secretory proteins/factors in the mosquito saliva creates an immunologically compromised micro-environment that can have a profound effect on the transmission efficiency, pathogen establishment, and disease development [5–7]. The presence of *Ae.aegypti* saliva causes a differential host immune response to CHIKV infections in mice [6], suppresses recruitment of T cells to the initial bite site thus enhancing West Nile virus dissemination [8], suppresses antimicrobial peptides and IFNs thus enhancing Dengue virus (DENV) infection in human keratinocytes [9] and modulates Rift Valley Fever virus pathogenicity in mice [10]. To that end, several saliva proteins have been isolated that are facilitators of mosquito feeding, modulators of skin immunity and regulators of virus transmission and dissemination in the vertebrate host [11]. For example, the aegyptin protein isolated from *Ae.aegypti* saliva aids in blood feeding [12]. Another isolated putative 34 kDa protein modulates DENV infection in human keratinocytes via immunomodulation [13] and serine proteases in *Ae.aegypti* saliva facilitate DENV dissemination in mice [11]. These studies provide important information about the complex roles of salivary proteins in virus-host interactions however, other components of saliva and their functions have not been identified or characterized.

MicroRNAs (miRNAs) are short 18–24 nucleotide non-coding RNAs that regulate gene expression post-transcriptionally by binding to complementary regions mainly in the 3′ UTRs of targeted messenger RNAs. MicroRNA expression patterns have been profiled in mosquitoes of medical importance such as *Anopheles gambiae* [14], *Anopheles stephensi [15], Ae.aegypti* [16], *Ae. albopictus* [17], *Culex quinquefasciatus* [18] and *Anopheles anthropophagus* [19]. Functional studies of these mosquito miRNAs have demonstrated their role in blood digestion and egg development in *Ae. aegypti* [20], blood-meal induced miRNA expression for regulation of immune genes in *Ae. aegypti* [21] and *Ae. albopictus* [22], altered patterns of expression in *An. stephensi* post-blood feeding [23] and growth-stage specific expression in *An. anthropophagus* [19]. These miRNA expression profiles are altered in mosquitoes infected with parasites. For instance, the obligate endosymbiont, *Wolbachia pipientis*, regulates specific miRNA levels for maintenance of its life cycle in *Ae.aegypti* mosquitoes [24,25]. MicroRNA levels were also manipulated in *An.stephensi* [23] and *An. gambiae* [14] infected with *Plasmodium* and in *Ae.aegypti* infected with Dengue 2 [26]

While miRNAs have been detected and profiled from mosquito cell lines and mosquitoes, miRNA profiles in mosquito saliva have not been investigated. In the present study, we sought to detect and identify miRNAs in the saliva of *Ae.aegypti* and *Ae. albopictus* mosquitoes via deep sequencing. Furthermore, to investigate the effect of CHIKV infection on saliva miRNA expression profiles, deep sequencing was also performed on CHIKV-infected *Ae.aegypti* and *Ae.albopictus* saliva. A total of 103 mature miRNAs were discovered in *Ae.aegypti* and *Ae.albopictus* saliva. Seventy-two of the detected miRNAs aligned with previously identified miRNAs while 31 were potential novel miRNAs. Furthermore, 59 and 30 known miRNAs were upregulated in *Ae.aegypti* and *Ae. albopictus* CHIKV-infected saliva respectively indicating the possible functional importance of these miRNAs in CHIKV dissemination and transmission in the host.

## Methods

### Cells and viruses

African green monkey kidney (Vero) cells were maintained with Dulbecco’s Modified Eagle Medium (DMEM; Gibco, Carlsbad, CA) and baby hamster kidney (BHK-21) cells were maintained with Modified Eagle’s Medium (MEM; Gibco, Carlsbad, CA) supplemented with 10% fetal bovine serum (FBS; Gibco, Carlsbad, CA) and 5% penicillin/streptomycin (P/S; 100U/mL/ 100μg/mL, Gibco, Carlsbad, CA) at 37°C with 5% CO_2_. The *Aedes albopictus* (C6/ 36) cell line was maintained in Leibowitz’s media (Invitrogen) supplemented with 10% FBS and 5% P/S at 28°C without CO_2_. The *Aedes Ae.aegypti* (AAG-2) cell line was maintained in Schneider’s Insect Cell Media (Invitrogen) supplemented with 10% FBS and 5% P/S at 28°C without CO_2._ The infectious clone, CHIKV-LR 5′ GFP (CHIKV), used in this study has been described and characterized previously [27], and was provided by Dr. Stephen Higgs.

### Mosquitoes

The *Aedes aegypti* (Higgs White eye) strain and the *Aedes albopictus* (La Reunion) strain are well characterized and competent vectors for CHIKV and CHIK-LR 5′ GFP viruses [27]. Mosquitoes were reared as previously described [16] within the UTMB insectary services core facility. Both species of mosquitoes were maintained at 28°C at a 14:10 hour (L: D) photoperiod with 10% sucrose solution provided *ad libitum.* Three to five day old females were used for all intrathoracic inoculations.

### Intrathoracic inoculations

Three to 5 day-old *Ae.aegypti* and *Ae. albopictus* mosquitoes were cold-anesthetized and intrathoracically inoculated with an approximately 0.1 μL inoculum of CHIKV-LR 5′ GFP: 4.6 TCID_50_/ mL. One hundred mosquitoes were inoculated per species after which inoculated mosquitoes were placed in 1 pint cartons in a 28°C incubator with 10% sucrose supplied *ad libitum* and a 14:10 hour (L:D) photoperiod. After 10 days post-infection (d.p.i), 50 infected and 50 uninfected mosquitoes were collected for each species, cold-anesthetized and saliva was collected. Briefly, saliva was collected by inserting each mosquito proboscis in a capillary tube with approximately 10μL of immersion oil and letting each mosquito salivate for 30 minutes at room temperature. Saliva were pooled according to infection status and species of mosquito, mixed with 250μL of DMEM and stored at −80°C until further processing.

### RNA extractions

The miRNeasy Kit (Qiagen, Valenica, CA) was used for extraction of microRNAs from the mosquito saliva. Briefly, 250μL of Trizol LS (Invitrogen, Carlsbad, CA) was added to the pooled mosquito saliva samples for virus inactivation and incubated overnight at −20°C. After 24 hours post-inactivation, RNA samples were thawed and 150μL of chloroform was added to each tube and shaken vigorously for 30 seconds. The samples were centrifuged for 15 minutes at 10000 × g at 4°C after which the clear, top layer was transferred to a new tube for total RNA and miRNA extraction using the Qiagen RNeasy extraction kit and Qiagen microRNA extraction kit respectively.

### Next generation sequencing

The Illumina TruSeq SmallRNA kit was used to prepare libraries of the microRNA samples. Briefly, short unique adapters were ligated to the 5′ and 3′ ends of short RNAs. Reverse-transcriptase and PCR were used to add the full length adapters required for Illumina sequencing, followed by gel purification of the correct size templates. The samples were tracked using “index tags” incorporated into the adapters. Library quality was evaluated using an Agilent DNA-1000 chip on an Agilent 2100 Bioanalyzer. Quantification of library DNA templates was performed using qPCR and a known-size reference standard.

### Sequence analysis

Cluster formation of the library DNA templates was performed using the TruSeq PE Cluster Kit v3 (Illumina) and the Illumina cBot workstation using conditions recommended by the manufacturer. Template input was adjusted to obtain a cluster density of 700–850 K/mm^2^. 50 base sequencing by synthesis was performed using TruSeq SBS kit v3 (Illumina) on an Illumina HiSeq 1000 using protocols defined by the manufacturer.

### Data analysis

The miRDeep2 software package [28] identified potential miRNA precursors by scanning for pileups of short reads in the genome alignment data. The region surrounding the pileup was excised computationally and analyzed for miRNA features. The structure of the potential precursor RNA was analyzed by RNAfold to determine the predicted secondary structure of the region and that structure was compared to typical miRNA precursor structures. If a likely structure was found, reads mapped to the precursor were counted and analyzed for the presence of mature and star miRNA sequences and then compared to the level of background sequences. The miRDeep2 algorithm used these results to score the likelihood that the predicted miRNA was real. The number of reads for each unique sequence was tracked. Following the miRDeep2 workflow the microRNAs were then compared against known microRNAs from the miRBase database (Version 20) with *Aedes aegypti* (AaegL1) as the reference species and *Anopheles gambiae* (AgamP3) as a related species. As the *Ae.albopictus* genome sequence was unavailable and miRNAs are highly conserved between species, reads from *Ae.albopictus* saliva were compared to known *Ae. aegypti* and *An. gambiae* miRNAs from miRBase database (Version 20). Novel microRNAs were identified by mapping the reads to the *Ae. aegypti* genome (AaegL2 from VectorBase VB-2014-02). Finally a table of known and potentially novel miRNAs was output with mapped read counts for each. Relative abundance of miRNAs in CHIKV infected samples were calculated by normalizing the data by tags per million (TPM) reads of total RNA as described previously [29].

### MicroRNA inhibition assay

MicroRNA inhibitors were designed based on the sequences of the following select microRNAs, aae-mir-12, aae-mir-125, aae-mir184, aar-mir-375, aae-mir-2490 and a control inhibitor with random sequence, Scramble, that was designed based on a previous study [30]. All miRNA inhibitors (MIR-12, MIR-125, MIR-184, MIR375 and MIR-2490) were synthesized by Integrated DNA Technologies^©^. The microRNAs that were chosen for this miRNA inhibition study were selected based on relative abundance levels of CHIKV- infected saliva, as well as, previous reports indicating their importance in modulating DENV and *Wolbachia* replication [21,24,31,32]. However, they have not been studied in the context of CHIKV replication. Additionally, these miRNAs have been identified and characterized in both AAG-2 and C6/36 mosquito cell lines [21,24,25,32,33]. Baby hamster kidney cells were used for this study as they are a fibroblast cell line and CHIKV targets and replicates in fibroblast cells in a natural infection [34,35]. The cell lines, AAG-2, BHK-21 and C6/36 cells, were grown to confluency and transfected in triplicate with 100 nanograms of each miRNA inhibitor via Cellfectin transfection reagent. As a control, cells were mock transfected without template. Cells were re-transfected at 48 hours post-transfection and were infected with CHIKV at a multiplicity of infection of 0.01 at 72 hours after initial transfection. As a control, mock transfected cells were also infected with CHIKV. Daily timepoints of 50μL were collected from each replicate until 72 hours post-infection, added to 450μL of diluent and stored at −80°C until further processing. A standard plaque assay on Vero cells was used to determine CHIKV titer at each timepoint as previously described [36].

### Statistical analysis

A 2-tailed student’s T-test (α 0.05) was used to analyze the significance of viral titer differences in the miRNA inhibition assay at each time point.

## Results

### Small RNA sequencing of *Aedes* spp. saliva

**Figure 1:**
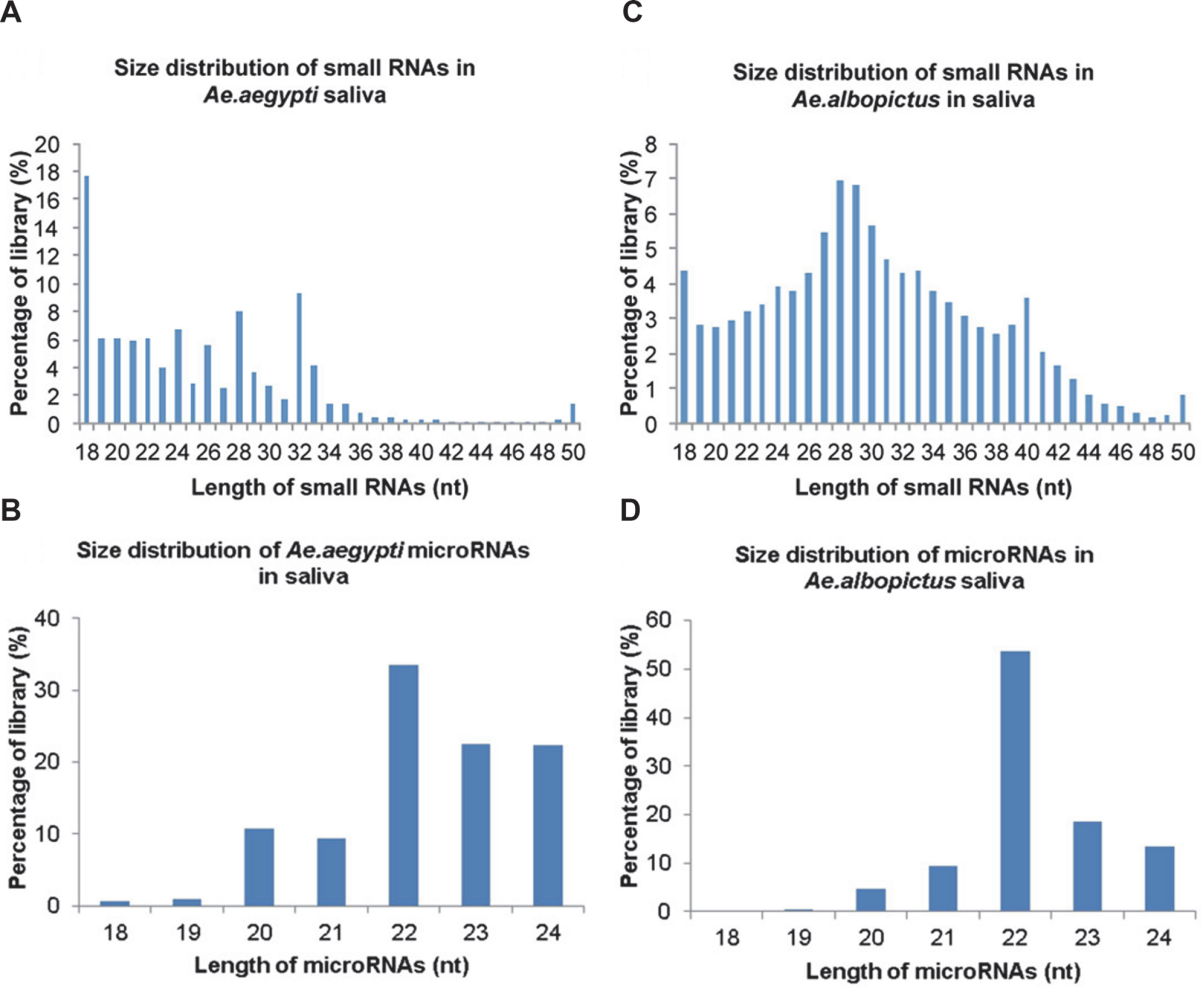
Identification of microRNAs in *Aedes* spp. saliva. *Aedes aegypti* and *Aedes albopictus* were intra-thoracically infected with Chikungunya virus. At 10 days post infection, saliva was collected from both infected and uninfected mosquitoes. Small RNAs were extracted from the saliva and subjected to deep sequencing, small RNA libraries were created and mapped to *Ae.aegypti* and *An.gambiae* miRNA databases. Figure a) size distribution and percentages of small RNAs in *Ae.aegypti* saliva, b) percentages of 18–24 nucleotide microRNAs in *Ae.aegypti* saliva library, c) size distribution and percentages of small RNAs in *Ae. albopictus* saliva, d) percentages of 18–24 nucleotide microRNAs in *Ae. albopictus* saliva library.

Small RNAs were extracted from the saliva of uninfected *Ae.aegypti* and *Ae.albopictus* mosquitoes and *Ae.aegypti* and *Ae. albopictus* mosquitoes infected with CHIKV. These small RNAs were then sequenced via Illumina-based high-throughput sequencing in order to identify small non-coding RNAs. A total of 14 × 10^6^ small RNAs were detected in *Ae.aegypti* saliva with a predominant size distribution of 18–33 nucleotides (nt) (Fig. 1A). Out of these, 18–24mers represented 56% of the library where 18mers represented a higher percentage of the library at 19% (Fig. 1A). After these RNAs were aligned with the *Ae.aegypti* genome, 43% of the *Ae.aegypti* library was composed of 18–24mers with 22mers exhibiting the highest frequency of reads (Fig. 1B). In comparison, small RNA sequencing of *Ae.albopictus* saliva, detected 3 × 10^6^ small RNAs and demonstrated a larger size range of 18–40 nts out of which 21% were represented by 18–24mers (Fig. 1C). *Ae.albopictus* saliva small miRNAs were matched to known *Ae.aegypti* and *An.gambiae* miRNAs and demonstrated a 48.8% representation of 18–24mers with 22mers having the highest frequency of reads (Fig. 1D).

In order to confirm that the detected 18–24mers were indeed mature miRNAs, miRDeep2 software was utilized to identify potential miRNAs based on nucleotide length, star sequence, stem-loop structural folding and sequence homology to already established reference genomes.Novel miRNAs were also identified using the criteria mentioned above. The stem-loop structures, the star sequence and premature miRNA sequence are shown for a select few novel miRNAs including miR-aae-249, miR-aae-23, miR-aal-43b and miR-aal-5. (S1 Fig.). Thirty-two percent of the detected miRNAs aligned to insect and mammal-specific miRNAs. Twenty-five percent were insect-specific and 11% were mosquito-specific miRNAs. Notably, 31% of the detected saliva miRNAs did not align with any known *Ae.aegypti* and *An.gambiae* miRNAs and were therefore designated as novel *Ae.aegypti* and *Ae.albopictus* miRNAs. Taken together these data provide strong evidence for the presence of mature insect miRNAs in *Ae.aegypti and Ae.albopictus* saliva.

### Identification of *Ae.aegypti* saliva miRNAs

After aligning the sequencing reads from *Ae.aegypti* saliva to the *Ae.aegypti* miRNA database, 72 distinct known miRNAs were identified in both uninfected and CHIKV-infected mosquito saliva (Table 1). In uninfected *Ae.aegypti* saliva, a total of 298283 reads were obtained with 283197 reads aligning with known miRNAs and 15086 reads that were novel miRNAs. In comparison, the total read count in CHIKV-infected *Ae.aegypti* saliva was 305894 reads with 251277 known miRNA reads and 54617 novel miRNA reads). The highest expressing miRNA in uninfected *Ae.aegypti* saliva was aae-mir-281-2-5p at 80151 reads. The other highly expressed miRNAs in uninfected *Ae.aegypti* saliva were aae-mir-281 (56394), aae-mir-2940 (25307), aae-mir-8 (47613), aae-mir-184 (10105) aae-mir-bantam (9969), aae-mir-263a (9084) and aae-mir125 (5863) (Table 1). Similarly, the highest expressing miRNAs in CHIKV-infected *Ae.aegypti* saliva were aae-mir-8 (50004), aae-mir-2940 (21514), aae-mir-263a (20584), aae-mir-bantam (18002), aae-mir-125 (15735), aae-mir-100 (13160), aae-mir-14 (12958) and aae-mir-285 (10006) (Table 1).

**Table 1:** Read counts of individual microRNAs detected in uninfected and CHIKV-infected *Ae.aegypti* saliva.

**MicroRNA**	**Read counts**		**Difference between infected and uninfected**	
	**Infected**	**Uninfected**	**Difference**	**Fold Difference**
aae-mir-8	50004	47613	2391	1.2
aae-mir-2940	21514	25307	−3793	1.0
aae-mir-263a	20584	9084	11500	2.6
aae-bantam	18002	9969	8033	2.0
aae-mir-125	15735	5863	9872	3.0
aae-mir-281	5833	56394	−50561	0.1
aae-mir-281-2-5p	9818	80151	−70333	0.1
aae-mir-100	13160	4309	8851	3.4
aae-mir-14	12958	5389	7569	2.7
aae-mir-285	10006	31	9975	363.8
aae-mir-276-1	9384	2584	6800	4.1
aae-mir-276-2	9291	2570	6721	4.1
aae-mir-317-1	6263	3367	2896	2.1
aae-mir-317-2	6263	3367	2896	2.1
aae-mir-184	5080	10105	−5025	0.6
aae-mir-12	4288	537	3751	9.0
aae-mir-277	3210	798	2412	4.5
aae-mir-10	2815	2035	780	1.6
aae-mir-279	2315	587	1728	4.4
aae-mir-2a	1695	580	1115	3.3
aae-mir-11	1650	2309	−659	0.8
aae-mir-1891-2	1576	1078	498	1.6
aae-mir-1891-1	1576	1078	498	1.6
aae-mir-1889	1195	298	897	4.5
aae-mir-2c	1133	313	820	4.1
aae-mir-210	1080	28	1052	43.5
aae-mir-34	1036	790	246	1.5
aae-mir-2b	905	219	686	4.7
aae-mir-92a	891	227	664	4.4
aae-mir-306	866	419	447	2.3
aae-mir-927	771	116	655	7.5
aae-mir-71	764	352	412	2.4
aae-mir-275	735	271	464	3.1
aae-mir-92b	700	430	270	1.8
aae-mir-996	693	169	524	4.6
aae-let-7	665	616	49	1.22
aae-mir-305	575	111	464	5.8
aae-mir-970	543	259	284	2.4
aae-mir-957	533	43	490	14.0
aae-mir-999	478	144	334	3.7
aae-mir-252	410	340	70	1.4
aae-mir-13	409	67	342	6.9
aae-mir-9c	332	299	33	1.3
aae-mir-980	330	18	312	20.7
aae-mir-133	277	11	266	28.4
aae-mir-1000-2	226	2	224	127.4
aae-mir-1000-1	226	2	224	127.4
aae-mir-998	211	140	71	1.7
aae-mir-190	190	36	154	5.9
aae-mir-308	172	44	128	4.4
aae-mir-307	161	0	161	161.0
aae-mir-315	146	10	136	16.5
aae-mir-263b	133	7	126	21.4
aae-mir-9a-2	112	67	45	1.9
aae-mir-9a-1	112	67	45	1.9
aae-mir-1890	109	33	76	3.7
aae-mir-932	109	50	59	2.5
aae-mir-2941-2	104	177	−73	0.7
aae-mir-2941-1	100	168	−68	0.7
aae-mir-87	98	34	64	3.2
aae-mir-2945	96	21	75	5.2
aae-mir-278	91	80	11	1.3
aae-mir-33	86	11	75	8.8
aae-mir-31	74	11	63	7.6
aae-mir-981	73	10	63	8.2
aae-mir-989	72	616	−544	0.1
aae-mir-2946	68	234	−166	0.3
aae-mir-375	54	189	−135	0.3
aae-mir-283	49	225	−176	0.2
aae-mir-9b	34	64	−30	0.6
aae-mir-1174	18	158	−140	0.1
aae-mir-1175	12	96	−84	0.1

### Detection of novel *Ae.aegypti* saliva miRNAs

Thirty-one novel mature miRNAs were detected from *Ae.aegypti* saliva after the predicted miRNAs were compared to the *Ae.aegypti* miRNA database and AaegL2 (Table 2). The highest expressed novel miRNA was aae-mir-143 with a count of 4275 and with a seed sequence match to aga-mir-14 (Table 2). Aae-mir-249, aae-mir-80 and aae-mir-5 were also highly expressed novel miRNAs in uninfected *Ae.aegypti* saliva with counts of 5385, 3773 and 2566 respectively (Table 2).

**Table 2:** Read counts of individual novel microRNAs detected in uninfected and CHIKV-infected *Ae.aegypti* saliva.

**Assigned Name**	**Consensus sequence**	**Read Counts**		**Difference between infected and uninfected**	
		**Infected**	**Uninfected**	**Difference**	**Fold Difference**
aae-mir-143	aacccguagauccgaacuugug	13129	4275	8854	0.8
aae-mir-249	ucagucuuuuucucucuccu	12951	5385	7566	0.7
aae-mir-5	uaggaacuucauaccgugcucu	9219	2566	6653	1.0
aae-mir-229	ucauaagacacacgcggcuau	544	259	285	0.6
aae-mir-778	uugguccccuucaaccagcugu	278	11	267	7.0
aae-mir-620	auuagaauguggaaucuguuuu	51	3	48	4.7
aae-mir-3069	uuuguucguuuggcucgagu	54	188	−134	0.1
aae-mir744	caucacagucugaguucuugcu	1451	1783	−332	0.2
aae-mir-115	ugugaugugacguagugguac	71	616	−545	0.0
aae-mir-23	uagcaccauucgaaaucaguac	10021	0	10021	10021.0
aae-mir-576	ggggauguagcucagugguagag	2033	0	2033	2033.0
aae-mir-320	uuucggauauguuuuagaaauuc	1262	0	1262	1262.0
aae-mir-214	uucccggacgagccccca	606	433	173	0.4
aae-mir-402	uucccggacgagccccca	606	433	173	0.4
aae-mir-65	ugcacacgacucgaugggauagac	397	0	397	397.0
aae-mir-341	gcaggaucguaggaggcu	294	0	294	294.0
aae-mir-3	agggucggagguucgaauccc	250	0	250	250.0
aae-mir-3798	auauuguccugucacagcag	226	0	226	226.0
aae-mir-187	auauuguccugucacagcag	226	0	226	160.0
aae-mir-242	caucgaucgcgcaccuga	160	0	160	139.0
aae-mir-309	guaggccggcggaaacuacuugc	139	21	118	1.8
aae-mir-210	uuuaccauuucaagaugacc	129	0	129	129.0
aae-mir-1571	gagaggccuguguaaucu	88	0	88	88.0
aae-mir-1247	guucgacucccagucggu	81	12	69	1.9
aae-mir-40	uugcguugauuaaguccc	81	0	81	81.0
aae-mir-843a	guccugucacggucgcca	74	0	74	81.0
aae-mir-117	uagcagaauccugaguaggac	73	0	73	74.0
aae-mir-360	gugagcaaauuuucaggugugu	67	0	67	73.0
aae-mir-359	guaacugacgcugaggag	56	0	56	67.0
aae-mir-80	auucucuguucguccacca	0	3773	−3773	0.0
aae-mir-109	aucacgucggggucacca	0	227	−227	0.0

### Identification of *Ae.albopictus* saliva miRNAs

A total of 43 miRNAs were identified in *Ae.albopictus* saliva. In uninfected *Ae.albopictus* saliva, a total of 12075 reads were obtained with 9180 reads aligning with known miRNAs and 4741 reads that were novel miRNAs (Table 3). In contrast, the total read count was 2-fold higher in CHIKV-infected *Ae.albopictus* saliva with a total count of 32593 reads with 16050 known miRNA reads and 16543 novel miRNA reads. Twenty-eight known miRNAs were identified in *Ae.albopictus* saliva (Table 3). The highest expressed miRNA in uninfected *Ae.albopictus* saliva was aae-mir-8 with a count of 12874 followed by aae-mir-2940 (2574), aae-mir-bantam (2127) and aae-mir-125 (2132) (Table 3). Highest read counts in CHIKV-infected *Ae.albopictus* saliva were from aae-mir-125 (4333), aae-mir-263a (4293), aae-mir-8 (2609), aae-mir-184 (2332) and aae-mir-100 (2255) (Table 3). With the exception of aae-mir-8, these miRNAs were upregulated at least 1.3-fold or higher in comparison with uninfected saliva (Table 3).

**Table 3:** Read counts of individual microRNAs detected in uninfected and CHIKV-infected *Ae.albopictus* saliva.

**MicroRNAs**	**Read Counts**		**Difference between Infected and Uninfected**	
	**Infected**	**Uninfected**	**Difference**	**Fold difference**
aae-mir-125	4333	2132	2201	2.2
aae-mir-263a	4293	1343	2950	3.4
aae-mir-8	2609	12874	−10265	0.2
aae-mir-184	2332	1885	447	1.3
aae-mir-100	2255	1204	1051	2.0
aae-mir-2940	1923	2574	−651	0.8
aae-mir-281	377	210	167	1.9
aae-mir-281-2-5p	752	292	460	2.7
aae-bantam	1116	2127	−1011	0.6
aae-mir-276-1	1046	482	564	2.3
aae-mir-276-2	1038	482	556	2.3
aae-mir-14	1032	731	301	1.5
aae-mir-10	887	153	734	6.2
aae-mir-927	795	151	644	5.6
aae-mir-317-1	632	844	−212	0.8
aae-mir-317-2	632	844	−212	0.8
aae-mir-277	430	212	218	2.2
aae-let-7	423	195	228	2.3
aae-mir-999	409	146	263	3.0
aae-mir-11	385	381	4	1.1
aae-mir-957	361	107	254	3.6
aae-mir-34	339	619	−280	0.6
aae-mir-92b	304	45	259	7.2
aae-mir-275	276	109	167	2.7
aae-mir-315	273	16	257	18.1
aae-mir-2a	212	171	41	1.3
aae-mir-2c	182	143	39	1.4
aae-mir-2b	148	147	1	1.069
aae-mir-12	145	132	13	1.2
aae-mir-1891-2	120	41	79	3.1
aae-mir-1891-1	120	41	79	3.1
aae-mir-133	109	25	84	4.6
aae-mir-252	105	202	−97	0.6
aae-mir-970	98	95	3	1.1
aae-mir-306	88	79	9	1.2
aae-mir-71	85	119	−34	0.8
aae-mir-279	46	131	−85	0.4
aae-mir-190	40	54	−14	0.8
aae-mir-305	35	55	−20	0.7
aae-mir-996	24	77	−53	0.3
aae-mir-210	18	808	−790	0.0
aae-mir-932	10	141	−131	0.1
aae-mir-285	1	114	−113	0.0

### Identification of novel miRNAs in *Ae.albopictus* saliva

Twenty-four novel, mature miRNAs were detected in *Ae.albopictus* saliva (Table 4). The highest expressing miRNA in uninfected *Ae.albopictus* saliva was aal-mir-43b which had a read count of 2134 followed by aal-mir-13 and aal-mir-43a at 1874 and 1200 reads respectively (Table 4). Highly expressed miRNAs in CHIKV-infected *Ae.albopictus* saliva were aal-mir-43b, aal-mir-43a, aal-mir-413a, aal-mir-5 and aal-mir-249 with read counts of 4339, 2253, 1643, 1035 and 1032 respectively (Table 4).

**Table 4:** Read counts of individual novel microRNAs detected in uninfected and CHIKV-infected *Ae.albopictus* saliva.

**Assigned Name**	**Consensus sequence**	**Read Counts**		**Difference between infected and uninfected**	
		**Infected**	**Uninfected**	**Difference**	**Fold Difference**
aal-mir-43a	aacccguagauccgaacuugug	2253	1200	1053	0.88
aal-mir-5	uaggaacuucauaccgugcucu	1035	482	553	1.00
aal-mir-249	ucagucuuuuucucucuccu	1032	729	303	0.66
aal-mir-778	uugguccccuucaaccagcugu	108	25	83	2.02
aal-mir-774	caucacagucugaguucuugcu	378	336	42	0.53
aal-mir-229	ucauaagacacacgcggcuau	98	95	3	0.48
aal-mir-43b	ucccugagacccuaacuuguga	4339	2134	2205	0.95
aal-mir-413a	guucgaauccuguucugg	1643	0	1643	1643.00
aal-mir-305	guucgauucccguucgag	984	0	984	984.00
aal-mir-6	guucgaauccugguaaga	892	0	892	892.00
aal-mir-47	ggggauguagcucagugguagag	751	0	751	751.00
aal-mir-157	uguggcguaguugguaac	398	0	398	398.00
aal-mir-28	guggagcaguauggaagc	373	0	373	373.00
aal-mir-69	guggcguaauugguagac	267	0	267	267.00
aal-mir-137	aggucguggguucgaacccc	232	0	232	232.00
aal-mir-2308	ggucggugguucgaaucc	119	0	119	119.00
aal-mir-214	uucccggacgagccccca	117	201	−84	0.27
aal-mir-309	guaggccggcggaaacuacuugc	82	36	46	1.07
aal-mir-62	acgucaaaucaucauguc	80	103	−23	0.36
aal-mir-143	accccugaaggaguuuucggag	71	0	71	71.00
aal-mir-127	guagccagaggaagagaaa	61	0	61	61.00
aal-mir-446	ucaaaucuugucgcgccg	53	0	53	53.00
aal-mir-408	guucgaauccuagucggga	52	0	52	52.00
aal-mir-13	uggacggagaacugauaagggc	0	1874	−1874	0.00

### Relative abundance of miRNAs in CHIKV-infected saliva

In comparison with uninfected *Ae.aegypti* saliva, CHIKV-infected saliva miRNA reads were slightly lower out of which 251277 reads corresponded with previously identified *Ae. aegypti* miRNAs (Table 1) and 54617 reads were novel miRNAs (Table 2). The highly expressed miRNAs, aae-mir-bantam, aae-mir-263a, aae-mir-125 and aae-mir-285 were upregulated in CHIKV-infected *Ae.aegypti* saliva with counts of 18002 (2.0-fold), 20584 (2.6-fold), 15735 (3.0-fold) and 10006 (>100-fold) when compared with uninfected read counts (Table 1). The novel miRNAs also did not demonstrate a significant total fold difference between the uninfected and infected saliva total read counts but individual miRNAs demonstrated differential expression (Table 2). In comparison with uninfected reads, highly expressed aae-mir-23, aae-mir-576 and aae-mir-320 were upregulated in CHIKV-infected *Ae.aegypti* saliva (Table 2) however aae-mir-80 was highly expressed in uninfected saliva (3773) but undetected in infected saliva.

Similar to *Ae.aegypti*, aae-mir-8 was also highly expressed at 12874 reads in uninfected *Ae.albopictus* saliva but in contrast to *Ae.aegypti,* was detectable in CHIKV-infected *Ae.albopictus* saliva (Table 3). Aae-mir-2940 was also downregulated (0.8-fold) in CHIKV-infected *Ae.albopictus* saliva whereas aae-mir-125 (2.2-fold), aae-mir-263a (3.4-fold), aae-mir-184 (1.3-fold) and aae-mir-100 (2.0-fold) were all upregulated in comparison with uninfected *Ae.albopictus* saliva. The highly expressed novel miRNAs, aal-mir-43b (1-fold), aal-mir-43a (0.9-fold), aal-mir-413a (>100-fold), aal-mir-5 (1-fold) and aal-mir-249 (0.7-fold) were upregulated in CHIKV-infected *Ae.albopictus* saliva in comparison with uninfected *Ae.albopictus* saliva with the exception of aal-mir-413a, which was not detected in uninfected saliva at all (Table 4). MicroRNA aal-mir-13 was highly expressed in uninfected *Ae.albopictus* saliva but was undetected in CHIKV-infected saliva.

### *Aedes* spp. saliva miRNAs modulate viral replication in mosquito and mammalian cells

**Figure 2:**
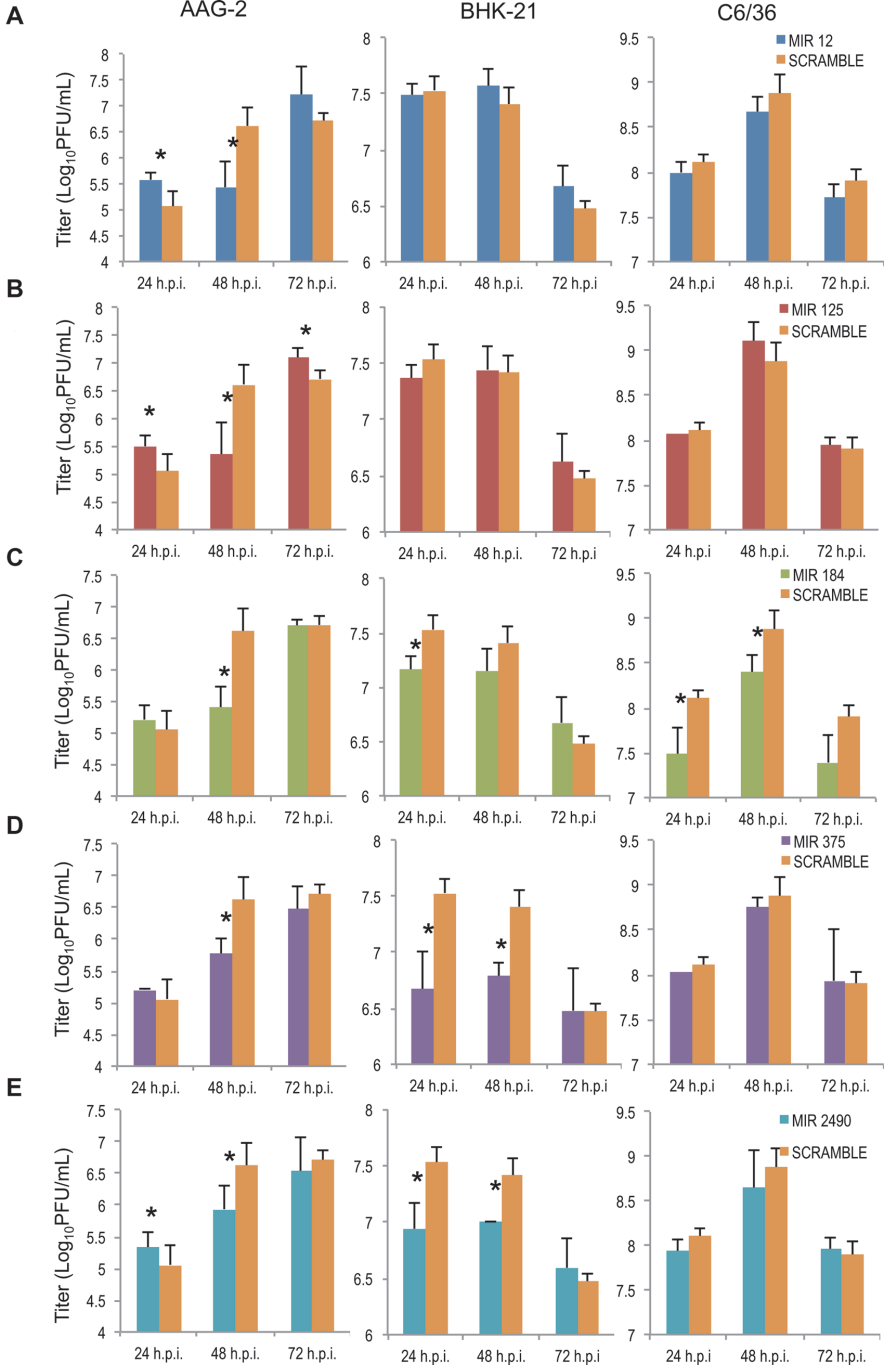
Saliva microRNAs regulate CHIKV replication in mosquito and mammalian cells. Mosquito (AAG-2 and C6/36) and mammalian (BHK-21) cells were transfected with miRNA inhibitors, a) MIR-12, b) MIR-125, c) MIR-184, d) MIR-375 and e) MIR-2940, and then infected with CHIKV at 72 hours post-transfection. Supernatant was collected daily for 72 hours and viral titers for each timepoint were determined via standard plaque assay on Vero cells.

In order to investigate the role of saliva miRNAs in the CHIKV replication, miRNA inhibitors were designed and transfected into mosquito (AAG-2 and C3/36) and mammalian (BHK-21) cells to profile CHIKV replication over time. In all three cell lines, there were no significant differences in CHIKV replication in non-transfected cells (CHIKV only), mock transfected cells (Transfected +CHIKV) and Scramble transfected cells. CHIKV replication in Scramble control cells peaked at 6.62 ± 0.03 log_10_PFU/mL at 48 hours post infection (h.p.i.) in AAG-2 cells. In Scramble BHK-21 cells and C6/36 cells, CHIKV peaked at 48 h.p.i. with a titer of 7.41 ± 0.15 and 8.88 ± 0.21 log_10_PFU/ mL, respectively. **AAG-2 cells:** At 24- 48 h.p.i., CHIKV titers were significantly lower (p < 0.05) in cells transfected with MIR-12 (Fig. 2A), MIR-125 (Fig. 2B) and mir-2490 (Fig. 2E) than in Scramble cells. At 48 h.p.i., CHIKV titers were significantly lower (p < 0.05) in cells transfected with MIR-184 (Fig. 2C) and MIR-375 (Fig. 2D). CHIKV titers peaked at 72 h.p.i. in AAG-2 cells transfected with miRNA inhibitors demonstrating an attenuated growth pattern compared to Scramble control cells where CHIKV titers peaked at 48 h.p.i. **BHK-21 cells:** Cells transfected with MIR-12 and MIR-125 did not exhibit any significant differences in CHIKV titers at any timepoint when compared with Scramble control cells. At 24 h.p.i., MIR-184 inhibited cells showed a significantly lower CHIKV titer of 7.16 ± 0.12 log_10_PFU/mL in comparison to 7.5 ± 1.2 log_10_PFU/mL (p<0.05). CHIKV titers were significantly lower (p<0.05) in MIR-375 and MIR-2940 inhibited cells at both 24 and 48 h.p.i. No significant viral titer differences were observed at 72 h.p.i. for any miRNA inhibitor. **C6/36 cells:** No significant differences were observed in titers for any miRNA inhibitor with the exception of MIR-184. At 24 and 48 h.p.i., CHIKV titers were 7.49 ± 0.29 and 8.40 ± 0.20 log_10_PFU/ mL respectively, which was significantly lower in comparison with Scramble control cells at those timepoints (p< 0.05).

## Discussion

MicroRNAs are generally considered to be intra-cellular. However circulating microRNAs have also been identified from human serum, saliva and other biofluids [37–40] but have not been described before in mosquito saliva. In the present study, mature microRNAs were discovered in the saliva of two species of *Aedes* spp. mosquitoes, *Ae.aegypti* and *Ae.albopictus*. To our knowledge, this is the first documentation of the presence of exogenous miRNAs in mosquito saliva where at least 70% of these miRNAs were found within the *Ae.aegypti* and related *Anopheles gambiae* known miRNA databases. These miRNAs were mosquito-specific, insect-specific or were both insect and mammal specific. Notably, 30% of these discovered miRNAs were not found in the known miRNA database and were designated novel mosquito miRNAs. Similar miRNAs were identified in both species of mosquitoes which corresponds with previous studies with *Ae.albopictus* and *Ae.aegypti* mosquito miRNAs [18] thus indicating the evolutionary pressure for miRNA sequence conservation and also potential multiple functions of each miRNA. Interestingly, the same miRNAs were highly expressed in both *Ae.albopictus* and *Ae.aegypti* saliva and these include aae-mir-8, aae-mir-2940, aae-mir-263a, aae-mir-bantam, aae-mir-125, aae-mir-184, aae-mir-281and aae-mir-100 all of which have been identified in *Aedes* spp. before [18].

Recent studies have shown exosomes to be the extracellular vesicles that transport miRNAs in biofluids like saliva and serum [37,41,42]. Microvesicles, such as exosomes, play a major role in intercellular communication and has been shown to transfer functional and intact proteins, lipids and nuclei acids between cells. The argonaute family of proteins has also been shown to transport miRNAs via serum [43]. Studies with Epstein-Barr virus (EBV) have demonstrated infected B cells releasing exosomes that contain EBV-miRNAs [44]. Therefore it is possible that exosomes or argonaute proteins are transporting miRNAs from the mosquito salivary glands to the bite site via saliva to potentially modulate viral replication.

The miR-184 was highly expressed in both species. High expression of miR-184 has been reported in other insects as well [18,45] where miR-184 is ubiquitously expressed in varying levels at all stages of *Drosophila* development [31]. In comparison with uninfected saliva, aae-mir-184 was highly expressed but downregulated in CHIKV infected *Ae.aegypti* saliva and upregulated in infected *Ae.albopictus* saliva. In our miRNA inhibition assays, CHIKV replication was inhibited in AAG-2 and BHK-21 cells at 48 and 24 h.p.i but not at 72 h.p.i. This corresponds with a previous study, where upregulation of miR-184 was observed in *S. frugiperda* cells after baculovirus infection at 24 h.p.i. but downregulated by 72 h.p.i. and could potentially explain the lack of CHIKV inhibition in our study at 72 h.p.i. [31]. Significant inhibition of CHIKV replication in both AAG-2 and BHK-21 cells also indicates the important role of miR-184 in arboviral infections in both mosquito and mammalian host. MicroRNA-184 has also been shown to increase in response to interleukin-22 (IL-22), a proinflammatory cytokine associated with inflammatory skin disorders, thereby reducing expression of Argonaute-2 (AGO 2) protein in human keratinocytes [46]. The AGO 2 protein recognizes and cleaves targeted dsRNA as part of the RNA-induced silencing complex (RISC) in the RNA interference (RNAi) pathway. As the RNAi pathway is an important defense pathway against viral infections in several mosquito species [47–50] differential expression of aae-miR-184 post-infection in mosquitoes could modulate AGO 2 levels thereby regulating viral replication at the initial site of infection. The C6/36 cell line has a dysfunctional RNAi pathway where Dicer-2, part of the RISC that associates with AGO 2, is lacking [51,52]. In the present study, CHIKV replication was inhibited at 24 and 48 h.p.i. in C6/36 cells suggesting a potentially more complex role of miR-184 in the RISC.

The highly expressed aae-miRNA-125 and aae-miR-100 were both upregulated in CHIKV-infected *Ae.aegypti* and *Ae.albopictus* saliva. MicroRNA-125, a homolog of *Drosophila* miR-let-7, is expressed in specific developmental stages of Drosophila [53]. MicroRNA-125, miR-100 and miR-let-7 are part of the same primary transcript and originate from a common genomic locus in *Drosophila* [54]. Additionally, clustering of the paralogs of these miRNAs also exists in the mouse genome suggesting multiple roles of these miRNAs across different species [55,56]. Target sites for mir-125a and mir-125b have been predicted to be within the 3′UTR of both mouse and human TNF-α transcripts [57] and miR-125b levels either increase or decrease in response to TNF-α stimulated macrophages both *in vitro* and *in vivo* [57]. Additionally, downregulation of TNFAIP results in increased levels of NF-κB which contributes to increased immune cell activity [58]. Therefore, both aae-mir-125 and aae-mir-100 could be contributing to regulating immune cell activity at the bite site in order to influence CHIKV replication.

The aae-miR-375 has been shown to be important in DENV replication [21] and was downregulated at least 34-fold in *Ae.aegypti* and undetected in *Ae.albopictus* in the present study. Predicted target sites for miR-375 include the *REL1* and *prohibitin*, the 5′UTR of *cactus*, the 3′UTR of *DEAD box ATP-dependent RNA helicase*, a hypothetical protein and the coding region of *kinesin* all of which showed significant modulation in response to *Ae.aegypti* mosquitoes injected with aae-miR-375 mimics [21]. *Cactus* and *REL1* regulate the Toll immune pathway and were differentially expressed in response to aae-miR-375 mimics in *Ae.aegypti* mosquitoes and AAG-2 cells [21]. Furthermore, presence of aae-miR-375 mimics increased DENV-2 levels in AAG-2 cells which corresponded with our miRNA inhibition assay where a decrease in CHIKV replication was observed in AAG-2 and BHK-21 cells after exposure to aae-miR-375 inhibitors. As the *cactus* gene inhibits NF-κB transcription factor activation, it seems that aae-miR-375 allows for enhanced virus infection in AAG-2 cells via downregulation of *cactus*. Indeed, DENV infection was attenuated when the *cactus* gene was silenced *Ae.aegypti* mosquitoes [59]. In another study, miR-375 function was enhanced by increased expression of AGO2 in mice suggesting a potential interaction of aae-miR-375 and AGO2 [60].

In AAG-2 cells and BHK-21 cells, aae-miR-2490 inhibitors significantly reduced CHIKV replication at 24 and 48 h.p.i. which corresponds with reduced *Wolbachia* replication in AAG-2 cells exposed to aae-miR-2490 inhibitors [25]. Additionally, the aae-miR-2940 has been shown to target and upregulate metalloprotease m41 ftsh expression in AAG-2 cells and *Ae.aegypti* mosquitoes after *Wolbachia* infection which enhances its replication [25]. In another study, aae-miR-2490-5p was shown to enhance West Nile virus replication in C6/36 cells but not aae-miR-2490-3p. In contrast, in our study, CHIKV replication was unaffected by aae-miR-2490-3p inhibition in C6/36 cells as the aae-miR-2490 inhibitor was designed against aae-miR-2490-3p due to the predominant number of read counts in the saliva (aae-miR-2490).

The aae-miR-12 was highly upregulated in CHIKV-infected *Ae.aegypti* saliva but was unaffected in *Ae.albopictus* saliva. In cells transfected with aae-miR-12 inhibitors, reduced CHIKV replication was observed in AAG-2 cells but not BHK-21 or C6/36 cells. While aae-miR-12 has not been characterized with viruses, a similar pattern was observed in AAG-2 cells inhibition of aae-miR-12 greatly reduced *Wolbachia* density [25]. Potential targets of aae-miR-12 were predicted to be *MCM6*(DNA replication licensing factor), *MCT1* (monocarboxylate transporter) and the *Exonuclease* gene however only *MCM6* and *MCT1* were down-regulated when exposed to aae-miR-12 mimics in AAG-2 cells [24].

Out of the 5 miRNAs inhibited, all demonstrated lower CHIKV titers in AAG-2 cells however, only miR-184, miR-375 and miR-2490, demonstrated decreased CHIKV titers in both mosquito (AAG-2) and mammalian (BHK-21) cells. This suggests a multiple roles and multiple target sites of these miRNAs across various species. It further suggests that these 5 miRNAs, along with the other highly expressed discovered miRNAs, could be acting in concert at the bite site to regulate viral replication, viral dissemination and immune cell activity in the host. Because inhibiting the miRNAs decreased viral replication, the presence of these miRNAs and upregulation of these miRNAs in mosquito saliva most likely enhances CHIKV replication and dissemination in the host and at the site of infection.

In conclusion, we have discovered microRNAs from mosquito saliva and have identified saliva miRNAs that are expressed only upon CHIKV infection. To our knowledge, this is the first report on the identification of exogenous mosquito saliva microRNAs. Identification of several miRNAs only in the CHIKV infected saliva suggests a possible importance in CHIKV transmission and establishment of infection in the host. Though the functional roles of these miRNAs are yet to be established, our *in-vitro* data from testing 5 miRNAs demonstrate their role in the regulation of CHIKV infection. These miRNAs may play an important role in regulating the establishment of CHIKV infection in the mammalian host during blood feeding, and are a subject of our future study.

## Supporting Information

S1 Fig Predicted stem-loop structures of novel microRNAs. The miRDeep2 software was used to confirm the presence of miRNAs based on nucleotide length, star sequence, stem-loop folding and homology to AaegL1 and AgamP3 genomes. Figures a) aae-miR-249 b) aae-miR-23 c) aal-miR-43b and d) aal-miR-5 show the predicted stem-loop structures, star and mature sequences of highly expressed novel microRNAs in *Ae.aegypti* and *Ae.albopictus* saliva. As the *Ae.albopictus* genome has not been described, aal-miR, was used to designate novel miRNAs identified in *Ae.albopictus.*
(TIFF)

## References

[1] Gratz NG (1999) Emerging and resurging vector-borne diseases. Annu Rev Entomol 44: 51–75. 9990716 10.1146/annurev.ento.44.1.51

[2] Gubler DJ (2002) The global emergence/resurgence of arboviral diseases as public health problems. Arch Med Res 33: 330–342. 12234522 10.1016/s0188-4409(02)00378-8

[3] Weaver SC (2014) Arrival of Chikungunya Virus in the New World: Prospects for Spread and Impact on Public Health. PLoS Negl Trop Dis 8: e2921 10.1371/journal.pntd.0002921 24967777 PMC4072586

[4] Higgs S (2014) Chikungunya Virus: A Major Emerging Threa. Vector-Borne and Zoonotic Diseases 14. 10.1089/vbz.2014.14.8.edit 25029622

[5] Schneider BS, Higgs S (2008) The enhancement of arbovirus transmission and disease by mosquito saliva is associated with modulation of the host immune response. Transactions of The Royal Society of Tropical Medicine and Hygiene 102: 400–408. 10.1016/j.trstmh.2008.01.024 18342898 PMC2561286

[6] Thangamani S, Higgs S, Ziegler S, Vanlandingham D, Tesh R, et al. (2010) Host Immune Response to Mosquito-Transmitted Chikungunya Virus Differs from That Elicited by Needle Inoculated Virus. PLoS ONE 5: e12137 10.1371/journal.pone.0012137 20711354 PMC2920837

[7] Styer LM, Lim P-Y, Louie KL, Albright RG, Kramer LD, et al. (2011) Mosquito Saliva Causes Enhancement of West Nile Virus Infection in Mice. Journal of Virology 85: 1517–1527. 21147918 10.1128/JVI.01112-10 PMC3028906

[8] Schneider BS, Soong L, Coffey LL, Stevenson HL, McGee CE, et al. (2010) *Aedes aegypti* Saliva Alters Leukocyte Recruitment and Cytokine Signaling by Antigen-Presenting Cells during West Nile Virus Infection. PLoS ONE 5: e11704 10.1371/journal.pone.0011704 20661470 PMC2908538

[9] Surasombatpattana P, Patramool S, Luplertlop N, Yssel H, Misse D (2012) *Aedes aegypti* Saliva Enhances Dengue Virus Infection of Human Keratinocytes by Suppressing Innate Immune Responses. J Invest Dermatol 132: 2103–2105. 10.1038/jid.2012.76 22475758

[10] Le Coupanec A, Babin D, Fiette L, Jouvion G, Ave P, et al. (2013) *Aedes* Mosquito Saliva Modulates Rift Valley Fever Virus Pathogenicity. PLoS Negl Trop Dis 7: e2237 10.1371/journal.pntd.0002237 23785528 PMC3681724

[11] Conway MJ, Watson AM, Colpitts TM, Dragovic SM, Li Z, et al. (2014) Mosquito Saliva Serine Protease Enhances Dissemination of Dengue Virus into the Mammalian Host. Journal of Virology 88: 164–175. 10.1128/JVI.02235-13 24131723 PMC3911723

[12] Calvo E, Tokumasu F, Marinotti O, Villeval J-L, Ribeiro JMC, et al. (2007) Aegyptin, a Novel Mosquito Salivary Gland Protein, Specifically Binds to Collagen and Prevents Its Interaction with Platelet Glycoprotein VI, Integrin α2β1, and von Willebrand Factor. Journal of Biological Chemistry 282: 26928–26938. 17650501 10.1074/jbc.M705669200 PMC2913440

[13] Surasombatpattana P, Ekchariyawat P, Hamel R, Patramool S, Thongrungkiat S, et al. (2014) *Aedes aegypti* Saliva Contains a Prominent 34-kDa Protein that Strongly Enhances Dengue Virus Replication in Human Keratinocytes. J Invest Dermatol 134: 281–284. 10.1038/jid.2013.251 23752041

[14] Winter F, Edaye S, Huttenhofer A, Brunel C (2007) *Anopheles gambiae* miRNAs as actors of defence reaction against *Plasmodium* invasion. Nucl Acids Res 35: 6953–6962. 17933784 10.1093/nar/gkm686 PMC2175301

[15] Mead E, Tu Z (2008) Cloning, characterization, and expression of microRNAs from the Asian malaria mosquito, *Anopheles stephensi* . BMC Genomics 9: 244 10.1186/1471-2164-9-244 18500992 PMC2430712

[16] Li S, Mead E, Liang S, Tu Z (2009) Direct sequencing and expression analysis of a large number of miRNAs in *Aedes aegypti* and a multi-species survey of novel mosquito miRNAs. BMC Genomics 10: 581 10.1186/1471-2164-10-581 19961592 PMC2797818

[17] Gu J, Hu W, Wu J, Zheng P, Chen M, et al. (2013) miRNA Genes of an Invasive Vector Mosquito, *Aedes albopictus* . PLoS ONE 8: e67638 10.1371/journal.pone.0067638 23840875 PMC3698096

[18] Skalsky R, Vanlandingham D, Scholle F, Higgs S, Cullen B (2010) Identification of microRNAs expressed in two mosquito vectors, *Aedes albopictus* and *Culex quinquefasciatus* . BMC Genomics 11: 119 10.1186/1471-2164-11-119 20167119 PMC2834634

[19] Liu W, Huang H, Xing C, Li C, Tan F, et al. (2014) Identification and characterization of the expression profile of microRNAs in *Anopheles anthropophagus* . Parasites & Vectors 7: 159 10.1186/1756-3305-7-159 24690438 PMC4022070

[20] Bryant B, Macdonald W, Raikhel AS (2010) microRNA miR-275 is indispensable for blood digestion and egg development in the mosquito *Aedes aegypti* . Proceedings of the National Academy of Sciences 107: 22391–22398. 10.1073/pnas.1016230107 21115818 PMC3012520

[21] Hussain M, Walker T, O’Neill SL, Asgari S (2013) Blood meal induced microRNA regulates development and immune associated genes in the Dengue mosquito vector, *Aedes aegypti* . Insect Biochemistry and Molecular Biology 43: 146–152. 10.1016/j.ibmb.2012.11.005 23202267

[22] Puthiyakunnon S, Yao Y, Li Y, Gu J, Peng H, et al. (2013) Functional characterization of three MicroRNAs of the Asian Tiger Mosquito, *Aedes albopictus* . Parasites & Vectors 6: 230 10.1186/1756-3305-6-230 23924583 PMC3750763

[23] Jain S, Rana V, Shrinet J, Sharma A, Tridibes A, et al. (2014) Blood Feeding and <Plasmodium Infection Alters the miRNome of *Anopheles stephensi* . PLoS ONE 9: e98402. 24866389 10.1371/journal.pone.0098402 PMC4035286

[24] Osei-Amo S, Hussain M, O’Neill SL, Asgari S (2012) *Wolbachia*-Induced aae-miR-12 miRNA Negatively Regulates the Expression of *MCT1* and *MCM6* Genes in *Wolbachia*-Infected Mosquito Cell Line. PLoS ONE 7: e50049 10.1371/journal.pone.0050049 23166816 PMC3500346

[25] Hussain M, Frentiu FD, Moreira LA, O’Neill SL, Asgari S (2011) Wolbachia uses host microRNAs to manipulate host gene expression and facilitate colonization of the dengue vector *Aedes aegypti* . Proceedings of the National Academy of Sciences 108: 9250–9255. 10.1073/pnas.1105469108 PMC3107320 21576469

[26] Campbell CL, Harrison T, Hess AM, Ebel GD (2014) MicroRNA levels are modulated in *Aedes aegypti* after exposure to Dengue-2. Insect Molecular Biology 23: 132–139. 10.1111/imb.12070 24237456 PMC4120961

[27] Tsetsarkin K, Higgs S, McGee CE, Lamballerie XD, Charrel RN, et al. (2006) Infectious Clones of Chikungunya Virus (La Réunion Isolate) for Vector Competence Studies. Vector-Borne and Zoonotic Diseases 6: 325–337. 17187566 10.1089/vbz.2006.6.325

[28] Friedlander M, Chen W, Adamidi C, Maaskola J, Einspanier R, et al. (2008) Discovering microRNAs from deep sequencing data using miRDeep. Nat Biotechnol 26: 407–415. 10.1038/nbt1394 18392026

[29] Shrinet J, Jain S, Jain J, Bhatnagar RK, Sunil S (2014) Next Generation Sequencing Reveals Regulation of Distinct *Aedes* microRNAs during Chikungunya Virus Development. PLoS Negl Trop Dis 8: e2616 10.1371/journal.pntd.0002616 24421911 PMC3888459

[30] Hussain M, Torres S, Schnettler E, Funk A, Grundhoff A, et al. (2012) West Nile virus encodes a microRNA-like small RNA in the 3′ untranslated region which up-regulates GATA4 mRNA and facilitates virus replication in mosquito cells. Nucleic Acids Research 40: 2210–2223. 10.1093/nar/gkr848 22080551 PMC3300009

[31] Mehrabadi M, Hussain M, Asgari S (2013) MicroRNAome of *Spodoptera frugiperda* cells (Sf9) and its alteration following baculovirus infection. Journal of General Virology 94: 1385–1397. 10.1099/vir.0.051060-0 23407421

[32] Slonchak A, Hussain M, Morales ST, Asgari S, Khromykh A (2014) Expression of mosquito microRNA aae-miR-2940-5p is down-regulated in response to West Nile Virus infection to restrict viral replication. Journal of Virology. 10.1128/JVI.00317-14 PMC4135961 24829359

[33] Zhang G, Hussain M, O’Neill SL, Asgari S (2013) *Wolbachia* uses a host microRNA to regulate transcripts of a methyltransferase, contributing to dengue virus inhibition in *Aedes aegypti* . Proceedings of the National Academy of Sciences 110: 10276–10281. 10.1073/pnas.1303603110 23733960 PMC3690878

[34] Tang BL (2012) The cell biology of Chikungunya virus infection. Cellular Microbiology 14: 1354–1363. 22686853 10.1111/j.1462-5822.2012.01825.x

[35] Selvamani SP, Mishra R, Singh SK (2014) Chikungunya Virus Exploits miR-146a to Regulate NF-κB Pathway in Human Synovial Fibroblasts. PLoS ONE 9: e103624 10.1371/journal.pone.0103624 25083878 PMC4118904

[36] Beaty BJ, Calisher CH, Shope RE (1995) Diagnostic procedures for viral, rickettsial, and chlamydial infections. In: Lennette EH, Lennette DA, Lennette ET, eds, editors. Arboviruses. Washinton D.C: American Public Health Association pp. 189–212.

[37] Gallo A, Alevizos I (2013) Isolation of Circulating MicroRNA in Saliva. In: Kosaka N, editor. Circulating MicroRNAs: Humana Press pp. 183–190.

[38] Hanson EK, Lubenow H, Ballantyne J (2009) Identification of forensically relevant body fluids using a panel of differentially expressed microRNAs. Analytical Biochemistry 387: 303–314. 10.1016/j.ab.2009.01.037 19454234

[39] Park NJ, Zhou H, Elashoff D, Henson BS, Kastratovic DA, et al. (2009) Salivary microRNA: Discovery, Characterization, and Clinical Utility for Oral Cancer Detection. Clinical Cancer Research 15: 5473–5477. 10.1158/1078-0432.CCR-09-0736 19706812 PMC2752355

[40] Weber JA, Baxter DH, Zhang S, Huang DY, How Huang K, et al. (2010) The MicroRNA Spectrum in 12 Body Fluids. Clinical Chemistry 56: 1733–1741. 10.1373/clinchem.2010.147405 20847327 PMC4846276

[41] Gallo A, Tandon M, Alevizos I, Illei GG (2012) The Majority of MicroRNAs Detectable in Serum and Saliva Is Concentrated in Exosomes. PLoS ONE 7: e30679 10.1371/journal.pone.0030679 22427800 PMC3302865

[42] Valadi H, Ekstrom K, Bossios A, Sjostrand M, Lee JJ, et al. (2007) Exosome-mediated transfer of mRNAs and microRNAs is a novel mechanism of genetic exchange between cells. Nat Cell Biol 9: 654–659. 17486113 10.1038/ncb1596

[43] Arroyo JD, Chevillet JR, Kroh EM, Ruf IK, Pritchard CC, et al. (2011) Argonaute2 complexes carry a population of circulating microRNAs independent of vesicles in human plasma. Proceedings of the National Academy of Sciences 108: 5003–5008. 10.1073/pnas.1019055108 21383194 PMC3064324

[44] Pegtel DM, van de Garde MDB, Middeldorp JM (2011) Viral miRNAs exploiting the endosomal—exosomal pathway for intercellular cross-talk and immune evasion. Biochimica et Biophysica Acta (BBA)—Gene Regulatory Mechanisms 1809: 715–721. 10.1016/j.bbagrm.2011.08.002 21855666

[45] Rao Z, He W, Liu L, Zheng S, Huang L, et al. (2012) Identification, Expression and Target Gene Analyses of MicroRNAs in *Spodoptera litura* . PLoS ONE 7: e37730 10.1371/journal.pone.0037730 22662202 PMC3360614

[46] Roberts JC, Warren RB, Griffiths CEM, Ross K (2013) Expression of microRNA-184 in keratinocytes represses argonaute 2. Journal of Cellular Physiology 228: 2314–2323. 10.1002/jcp.24401 23696368

[47] Blair CD (2011) Mosquito RNAi is the major innate immune pathway controlling arbovirus infection and transmission. Future Microbiology 6: 265–277. 10.2217/fmb.11.11 21449839 PMC3126673

[48] Brackney DE, Beane JE, Ebel GD (2009) RNAi Targeting of West Nile Virus in Mosquito Midguts Promotes Virus Diversification. PLoS Pathog 5: e1000502 10.1371/journal.ppat.1000502 19578437 PMC2698148

[49] Khoo C, Piper J, Sanchez-Vargas I, Olson K, Franz A (2010) The RNA interference pathway affects midgut infection- and escape barriers for Sindbis virus in *Aedes aegypti* . BMC Microbiology 10: 130 10.1186/1471-2180-10-130 20426860 PMC2877022

[50] Sánchez-Vargas I, Scott JC, Poole-Smith BK, Franz AWE, Barbosa-Solomieu V, et al. (2009) Dengue Virus Type 2 Infections of *Aedes aegypti* Are Modulated by the Mosquito’s RNA Interference Pathway. PLoS Pathog 5: e1000299 10.1371/journal.ppat.1000299 19214215 PMC2633610

[51] Brackney DE, Scott JC, Sagawa F, Woodward JE, Miller NA, et al. (2010) C6/36 *Aedes albopictus* Cells Have a Dysfunctional Antiviral RNA Interference Response. PLoS Neglected Tropical Diseases 4: e856 10.1371/journal.pntd.0000856 21049065 PMC2964293

[52] Scott JC, Brackney DE, Campbell CL, Bondu-Hawkins V, Hjelle B, et al. (2010) Comparison of Dengue Virus Type 2-Specific Small RNAs from RNA Interference-Competent and -Incompetent Mosquito Cells. PLoS Negl Trop Dis 4: e848 10.1371/journal.pntd.0000848 21049014 PMC2964303

[53] Caygill EE, Johnston LA (2008) Temporal Regulation of Metamorphic Processes in *Drosophila* by the let-7 and miR-125 Heterochronic MicroRNAs. Current Biology 18: 943–950. 10.1016/j.cub.2008.06.020 18571409 PMC2736146

[54] Bashirullah A, Pasquinelli AE, Kiger AA, Perrimon N, Ruvkun G, et al. (2003) Coordinate regulation of small temporal RNAs at the onset of Drosophila metamorphosis. Developmental Biology 259: 1–8. 12812783 10.1016/s0012-1606(03)00063-0

[55] Schulman BRM, Esquela-Kerscher A, Slack FJ (2005) Reciprocal expression of lin-41 and the microRNAs let-7 and mir-125 during mouse embryogenesis. Developmental Dynamics 234: 1046–1054. 16247770 10.1002/dvdy.20599 PMC2596717

[56] Griffiths-Jones S, Grocock RJ, van Dongen S, Bateman A, Enright AJ (2006) miRBase: microRNA sequences, targets and gene nomenclature. Nucleic Acids Research 34: D140–D144. 16381832 10.1093/nar/gkj112 PMC1347474

[57] Tili E, Michaille J-J, Cimino A, Costinean S, Dumitru CD, et al. (2007) Modulation of miR-155 and miR-125b Levels following Lipopolysaccharide/TNF-α Stimulation and Their Possible Roles in Regulating the Response to Endotoxin Shock. The Journal of Immunology 179: 5082–5089. 17911593 10.4049/jimmunol.179.8.5082

[58] Kim S-W, Ramasamy K, Bouamar H, Lin A-P, Jiang D, et al. (2012) MicroRNAs miR-125a and miR-125b constitutively activate the NF-κB pathway by targeting the tumor necrosis factor alpha-induced protein 3 (TNFAIP3, A20). Proceedings of the National Academy of Sciences 109: 7865–7870. 10.1073/pnas.1200081109 22550173 PMC3356650

[59] Xi Z, Ramirez JL, Dimopoulos G (2008) The *Aedes aegypti* Toll Pathway Controls Dengue Virus Infection. PLoS Pathog 4: e1000098 10.1371/journal.ppat.1000098 18604274 PMC2435278

[60] Tattikota SG, Rathjen T, McAnulty SJ, Wessels H-H, Akerman I, et al. (2014) Argonaute2 Mediates Compensatory Expansion of the Pancreatic β Cell. Cell Metabolism 19: 122–134. 10.1016/j.cmet.2013.11.015 24361012 PMC3945818

