# Supplementary figures and images for: Discovery of Mosquito Saliva MicroRNAs during CHIKV Infection
Source: PLoS Negl Trop Dis. 2015 Jan 22;9(1):e0003386. doi: 10.1371/journal.pntd.0003386 (PMC4303268; doi:10.1371/journal.pntd.0003386)

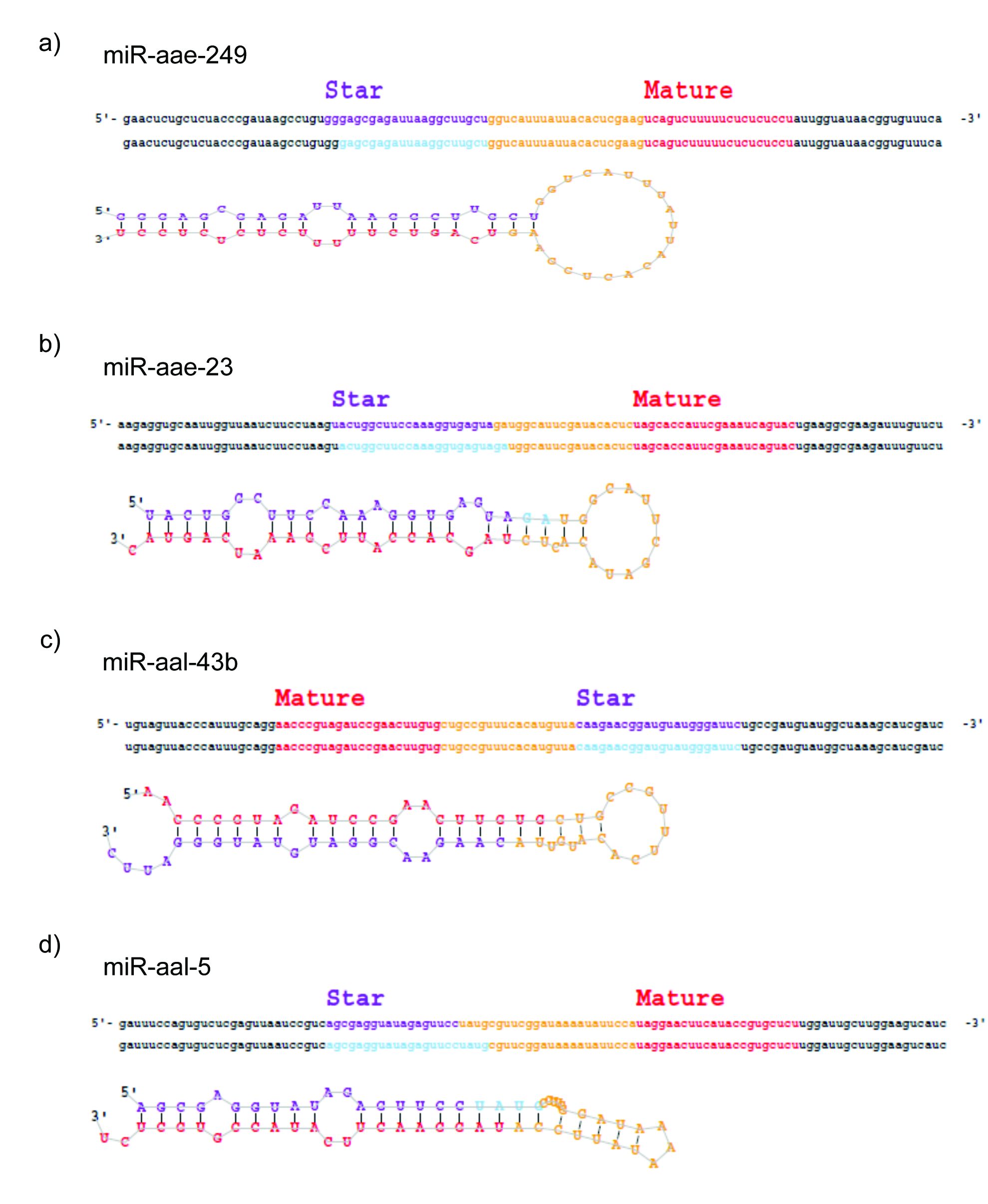

Supplement: S1 Fig — The miRDeep2 software was used to confirm the presence of miRNAs based on nucleotide length, star sequence, stem-loop folding and homology to AaegL1 and AgamP3 genomes. Figures a) aae-miR-249 b) aae-miR-23 c) aal-miR-43b and d) aal-miR-5 show the predicted stem-loop structures, star and mature sequences of highly expressed novel microRNAs in Ae.aegypti and Ae.albopictus saliva. As the Ae.albopictus genome has not been described, aal-miR, was used to designate novel miRNAs identified in Ae.albopictus. (TIFF) [file pntd.0003386.s001.tiff]
